# Prevalence and consequences of chromosomal abnormalities in Canadian commercial swine herds

**DOI:** 10.1186/s12711-016-0246-5

**Published:** 2016-09-12

**Authors:** Anh T. Quach, Tamas Revay, Daniel A. F. Villagomez, Mariana P. Macedo, Alison Sullivan, Laurence Maignel, Stefanie Wyss, Brian Sullivan, W. Allan King

**Affiliations:** 1 Department of Biomedical Sciences, University of Guelph, Guelph, ON Canada; 2 Canadian Centre for Swine Improvement Inc., Ottawa, ON Canada; 3 Departamento de Produccion Animal, Universidad de Guadalajara, Zapopan, Jalisco Mexico

## Abstract

**Background:**

Structural chromosome abnormalities are well known as factors that reduce fertility rate in domestic pigs. According to large-scale national cytogenetic screening programs that are implemented in France, it is estimated that new chromosome abnormalities occur at a rate of 0.5 % in fertility-unproven boars.

**Results:**

This work aimed at estimating the prevalence and consequences of chromosome abnormalities in commercial swine operations in Canada. We found pig carriers at a frequency of 1.64 % (12 out of 732 boars). Carrier pigs consistently showed lower fertility values. The total number of piglets born for litters from carrier boars was between 4 and 46 % lower than the herd average. Similarly, carrier boars produced litters with a total number of piglets born alive that was between 6 and 28 % lower than the herd average. A total of 12 new structural chromosome abnormalities were identified.

**Conclusions:**

Reproductive performance is significantly reduced in sires with chromosome abnormalities. The incidence of such abnormal sires appears relatively high in populations without routine cytogenetic screening such as observed for Canada in this study. Systematic cytogenetic screening of potential breeding boars would minimise the risk of carriers of chromosome aberrations entering artificial insemination centres. This would avoid the large negative effects on productivity for the commercial sow herds and reduce the risk of transmitting abnormalities to future generations in nucleus farms.

**Electronic supplementary material:**

The online version of this article (doi:10.1186/s12711-016-0246-5) contains supplementary material, which is available to authorized users.

## Background

Pork is the most consumed meat in the world [1, 2] and with the expansion of the global population, the need for efficient large-scale breeding is more important than ever. Factors such as fertility and litter size have major impacts on the economics of pork production [3]. Minimizing embryonic loss during pregnancy is key to improving litter size and it is well known that chromosome abnormalities are the major etiologic factors in the risk of embryo malformations and early embryo mortality in the domestic pig [4]. The negative impact of chromosome abnormalities on farm animal reproduction has led to the establishment of cytogenetic screening programs in many countries [5]. The largest of these programs was initiated in France over 20 years ago and led to the regular testing of all young artificial insemination (AI) boars in the country. This resulted in the most precise estimate of the prevalence of structural chromosomal abnormalities in a farm animal species, and in the appreciation of economic returns when the breeding companies cull carriers. The overall rate of chromosomal rearrangements in hypoprolific boars in service is close to 50 % and its prevalence in young untested AI boar candidates is 0.47 % [6]. This most probably reflects the rate of “de novo” abnormalities, since in other less intensively tested populations the frequency of abnormalities is usually much higher according to reports from Poland (1 %), the Netherlands (1.5 %) and Spain (3.4 %) [5, 7, 8]. Prior to this study, a systematical screening for chromosome abnormalities was not implemented in Canada but a few cases have been identified in Canadian pig populations, including a rcp(Xp+;14q−) [9, 10], rcp(1;6)(p22,q12), rcp(10;13), and rcp(9;14)(p24;q27) [11].

Therefore, this study aimed at investigating the prevalence and economic consequences of undetected chromosome abnormalities in commercial swine operations, which had not previously been screened.

## Methods

### Animals

Peripheral blood samples were collected from 732 unproven young boars raised on various Canadian farms. The tested population consisted of four breeds: Duroc (n = 340), Landrace (n = 222), Yorkshire (n = 146) and Pietrain (n = 24). In addition, karyotype analysis included the relatives and offspring of boars that were identified as carriers of chromosome aberrations where available (79 animals). These relatives were identified based on well-managed breeding records of genetic supplier farms.

### Chromosome analysis

Lymphocyte cultures from whole blood were set up according to standard cytogenetic methods, as previously described [11]. Images of GTG-banded [12] metaphase spreads were captured using a Leica DM5500B microscope (Leica), equipped with a Retiga Exi Fast (QImaging) digital camera and the OpenLab imaging software (Perkin Elmer). For each animal, 10 to 20 metaphase spreads were examined and at least three good quality metaphases per animal were karyotyped according to the international standard karyotype for the domestic pig [13], using the SmartType software (Digital Scientific UK).

Chromosome preparations that were prepared from Robertsonian translocation carriers were also stained for 5 min in 1 μg/mL propidium iodide in phosphate buffered saline buffer, covered with Vectashield, then denatured by placing them into a hot oven (90 °C) for 5 to 8 min till a C-banding-like pattern could be observed under the fluorescent microscope.

### Analysis of reproductive data and economic losses

Reproductive data for each identified carrier boar and its relatives (if available) were collected and compared with their herd averages. The latter was calculated for the period during which the carrier boar was in service. In addition, the direct boar effect on litter size (DBE) [14] was also used for comparison, when available. The DBE value precisely shows how many more or less piglets a given boar produces per litter on average, since the estimated boar effects are corrected for all identified environmental effects and breeding values of its mates. The Student’s t test was used to compare the available litter size related trait data, such as “total number of piglets born” (TNB), “total number of piglets born alive” (NBA), “number of stillborn piglets” (NSB) and “number of mummified piglets” (MUMM) for each translocation carrier boar with the corresponding data for the herd. The total loss in piglets per affected boar was calculated as the difference between the average TNB for the affected males subtracted from the average TNB for the herd multiplied by the number of litters born. The economic loss was calculated based on an average net weanling market price of $25 CDN per piglet [15]. We calculated that ~1.3 million sows are bred on average 2.2 times/year in Canada [16].

## Results

### Prevalence of chromosome abnormalities

Among the 732 karyotyped young Canadian AI boars, 12 were detected as carriers of a chromosomal abnormality, which represents a prevalence of 1.64 %. The 11 identified chromosome rearrangements fell into three different types of structural chromosome abnormalities including nine reciprocal translocations, one Robertsonian translocation and one inversion. Table 1 summarizes the cases and the identified chromosome abnormalities with their presumptive breakpoints based on the observed GTG-banding pattern. Table 2 provides a comparison to previously published chromosomal rearrangements in pig that involve the same chromosomes. Detailed description and representative karyotype images are in Additional file 1.

**Table 1:** Summary of the identified chromosome abnormalities

Case #	Abnormality	Breed	Origin
1	rcp(1;5)(q21;q23)	L	NA
2	rcp(1;15)(q211;q13)	Y	Probably maternal^a^
3	rcp(2;5)(p16;p11)	L	NA
4	rcp(3;4)(p15;q13)	L	Maternal
5	rcp(3;12)(p13;q15)	Y	Probably de novo^a^
6	rcp(6;7)(p15; q13)	D	De novo
7	rcp(7;15)(q13;q13)	D	NA
8	rcp(8;13)(p21;q41)	L	De novo
9	rcp(12;14)(q15;q23)	D	Maternal
10	Rob(13;17)	L	Maternal
11	inv(8)(q11;q25)	D	Probably maternal^a^

*rcp* reciprocal translocation, *inv* chromosome inversion, *Rob* Robersonian chromosome translocation (i.e. chromosome centric fusion), *L* Landrace, *Y* Yorkshire, *D* Duroc

^a^The probable inheritance was inferred from the DBE of relatives

**Table 2:** Comparison of cases from the current study to literature data where the same chromosomes are involved in the rearrangement

Rearrangements	Fragile sites	Breed	References
t(1;5)(q21;q23)	1q21	Landrace	This study (case 1)
t(1;5)(q21;q21)	1q21	Polish	[17]
t(1;15)(q211;q13)	1q211	Duroc	This study (case 2)
t(1;15)(p25;q13)	1p25	Landrace, Finnish	[18]
t(1;15)(q17;q22)	1q17	Synthetic	[6]
t(1;15)(q27;q26)	1q26	Large white	[19]
t(7;15)(q13;q13)		Duroc	This study (case 7)
t(7;15)(7q − ;15q +)		–	[20]
t(7;15)(q24;q12)		Large white	[21]
t(7;15)(q24;q26)		–	[22]
rcp(8;13)(p21;q41)		Landrace	This study (case 8)
t(8;13)(q27;q36)		–	[23]
t(12;14)(q15;q23)		Duroc	This study (case 9)
t(12,14)(q13;q15)	14q15	French Duroc	[6]
t(12;14)(q15;q13)		Pietrain	[6]
Inv(8)(q11;q25)		Duroc	This study (case 11)
Inv(8)(p21;q11)		Pietrain	[5]
Inv(8)(p11;q25)		Large white	[5]
Inv(8)(p11;p12)		Polish Landrace	[24]

### Reproductive performance of carriers of chromosome abnormalities

All boars carrying a chromosome abnormality were healthy with a normal phenotype and were already used or selected as potential candidates for breeding. Semen quality and quantity for the identified carrier boars had been tested on-farm and were within the average or above the range of the corresponding values of their herd.

Breeding and farrowing records showed a significant reduction in fertility for most of the cases (Table 3). The in-herd calculated TNB parameter for reciprocal translocation carriers varied from 6.8 to 12.7 piglets per litter, which represents 4 to 46 % less than their herd average. There was one carrier (case 6) that had a higher TNB but it was based on only four litters and the difference was not significant. The other registered fertility parameters also differed from their herd average, i.e. NBA was reduced by 6 to 28 %, NSB by 14 to 66 % and MUMM increased by 22 to 85 %. The NSB and MUMM-parameter values of the Rob(13;17) Robertsonian translocation carriers were slightly increased, although not significantly.

**Table 3:** Fertility data of carriers

Case #	# litters	Ave litter size	% TNB	% NBA	% NSB	% MUMM	DBE	Total piglet lost^a^	Cost (CAD)
1									
2	13	10.8	−27***	−28***	−14^ns^	+51^ns^	−4.82	52	1300
3	25	7.2	−46					153	3825
4	73	9.2	−22***	−21***	−66***	+85***	−2.67	189	8505
5	15	10.7	−17*	−17*	−21^ns^	+63***	−4.4	33	825
6	4	10	+7.5						
7	54	6	−36					182	4550
8	61	8.4	−28***	−27***	−38***	+22**	−4.39		
9	6	6.8	−27						
10	68	12.7	−4^ns^	−6^ns^	+19^ns^	+14^ns^			
11	N/A								

*** p < 0.001; ** p < 0.01; * p < 0.05

^ns^no statistical difference when compared with the same herd

^a^Calculated based on the minimal weanling cost of $25

The DBE on litter size, a parameter that estimates the phenotypic variation of the trait attributable directly to the sire was available for cases 2, 4, 5 and 8, i.e. −4.82, −2.67, −4.4 and −4.39, respectively, which indicated a strong adverse effect on litter size.

Regarding case 4, in addition to the boar, its dam, a full sister and two maternal half-sisters were also identified as carriers of rcp(3;4). The dam had given birth to six litters and the three sisters to seven litters in total. Average TNB, NBA, NSB and MUMM for carrier sows were equal to 11.5, 10.5, 1 and 0.38 piglets per litter, respectively. The TNB value represented a 10 % reduction compared with the average value of 12.8 piglets per litter for the herd. The NSB value was similar to the average value for the herd (1 vs. 0.9 piglets/litter) and MUMM was slightly lower (0.38 vs. 0.5 piglets/litter).

### Origin and dissemination of chromosome rearrangements

The parents of five carrier boars (cases 4, 6, 8, 9 and 10) were available for cytogenetic investigation. Maternal origin of the abnormality was found in three cases (cases 4, 9 and 10), while cases 6 and 8 were de novo events with both parents having a normal karyotype (Table 1). A full sister and three half-sisters of the rcp(3;4) carrier boar (case 4) were identified as translocation carriers as well.

Alternatively, if the parents are not available for testing, the DBE can provide information to infer the inheritance of the translocation and advise the owners for a follow-up testing strategy (Table 3). The Yorkshire boar (case 2), which carried a rcp(1;15), had a very low DBE (−4.82) and its sire and paternal grand-sire had highly negative DBE values as well (−6.03 from 19 litters and −4.7 from 63 litters, respectively), while the dam and maternal grandparents had average litter sizes. Although the origin of this abnormality could not be determined by direct karyotyping of the parents, the DBE values support a paternal origin. Similarly, the rcp(3;12) carrier boar (case 5) had a DBE value of −4.4 based on data from 11 litters. However, its parents and grandparents all had average litter sizes. Specifically, the sire had DBE of 0.82 (19 litters), the paternal grand-sire a DBE of −0.57 (94 litters), the maternal grand-sire a DBE of 0.452 (10 litters). Therefore, we inferred that rcp(3;12) occurred de novo. Moreover, the dam and maternal grand-sire of case 11 (inv(8)) had litter sizes that were less than the herd average, thus a maternal origin can be hypothesized. Specifically, its dam had 6.5 piglets per litter (across four litters; herd average was 9.64 piglets per litter) and its maternal grand-sire had a DBE of −2.78 (eight litters).

The origin of rcp(7;15) (case 7) could not be determined, since the sire and dam were no longer available and the tested full-sister and two paternal half-sisters were all normal.

Although most of the sampled boars were young and not in service, in three cases, the carriers had already produced offspring, which made it possible to study the dissemination of this abnormality among the progeny. Karyotyping of 23 randomly selected offspring (5 males and 18 females) of case 5 with rcp(3;12) led to the identification of seven carrier females (30 % transmission rate). There were two male and two female carriers of rcp(7;15) (case 7) among the 15 randomly selected offspring (7 males and 8 females), thus resulting in a very similar transmission rate of 27 %. These transmission rates were based on partial litters and were statistically different from the expected dissemination rate (50 %). In addition, on average 36 % of the piglets from two litters (9/25) (44 % (4) males, 56 % (5) females) were identified as carriers when a rob(13;17) carrier boar was mated with normal sows. When both parents were heterozygous carriers, 76 % (22/29) of the progeny inherited the translocation, among which 48 % were heterozygous (14/29) and 28 % (8/29) were homozygous carriers. Among the carrier piglets, 59 % (13/22) were females and 41 % (9/22) were males. These mating yielded surprisingly large litter sizes (13 and 16 piglets).

## Discussion

Pigs with chromosomal abnormalities in a balanced condition usually express normal physical characteristics and growth rate, thus, most often the phenotype does not provide information for their detection and prompt elimination from the herd [5, 25]. However, their reproductive efficiency is affected, i.e. due to the generation of genetically-unbalanced gametes at meiosis and concomitant early embryo loss, mating of such individuals often results in reduced litter size, and thus they are referred to as hypoprolific or subfertile animals [26]. The fact that almost 50 % of hypoprolific boars carry balanced chromosome rearrangements emphasizes that structural chromosome abnormalities are a major cause of reproductive failure in pigs [5, 11, 25, 27]. Although the average reduction in litter size of a sow mated with a carrier boar can range from 10 to 100 %, elimination of boar carriers before major breeding decisions is not an easy task at the farm level. Systematic cytogenetic screening of boars potentially used for AI is currently the most effective way of identifying boars that are carriers of chromosome abnormalities and at risk of producing small litters [5].

This survey involved six large commercial swine operations, each with several subsidiary farms, and resulted in the identification of nine reciprocal translocations and one inversion among 732 pigs. All represent novel cases of chromosomal abnormalities that were not previously reported. Table 2 compares these newly identified chromosome abnormalities to published cases involving the same pig chromosomes. The fact that the abnormalities were not detected in multiple herds suggests lack of inbreeding in the pig population sampled or random distribution of founder animals. Nevertheless, the single Robertsonian translocation (Rob(13,17)) that we detected in a Canadian herd, has already been reported in pigs from several countries including China, France, Germany and Mexico[6, 28–30].

The estimated prevalence of chromosome abnormalities in the Canadian pig populations (1.64 %) appears to be much higher than other frequencies recently reported in other countries. For instance, a prevalence of 0.5 % was estimated in France, although in this country systematical karyotype analysis and selective elimination from breeding populations have been in place for over two decades [5], thereby effectively eliminating all but de novo occurrences. At present, it is not known if genetic predispositions for de novo chromosome abnormalities occur in certain breeds of pigs or whether a specific genotype of an individual makes it susceptible for chromosome mutations to occur. Such information would provide an extra tool for breeding decisions and early elimination of undesirable animals from breeding programs.

In addition to determining the prevalence of chromosome abnormalities in commercial swine operations in Canada, our aim was to investigate the effect of the rearrangements on the overall fertility of carriers. For nine of the 12 carrier boars identified, comprehensive data on production parameters was available for analysis. For all nine boars, standard reproductive parameters such as TNB and NBA were reduced compared with the herd averages of the same farm. Both of these parameters are considered as crucial animal production parameters [31, 32]. The extent of fertility reduction varied between 4 and 46 % depending on the chromosomal rearrangement carried by the animal with reciprocal translocations causing the most adverse effects on TNB (−17 to −46 %, respectively). Studies on the Rob(13;17) translocation showed a reduction of fertility between 10 and 20 % [33]. However, in our study, boars carrying the Rob(13;17) translocation had only a slight reduction in TNB (4 %) which is in agreement with the findings of Pinton et al. [34] who reported 2.96 to 3.83 % of unbalanced spermatozoa by FISH (fluorescent in situ hybridization) analysis. Because the Rob(13;17) translocation resulted in only a minor reduction in litter size, litter size parameters did not allow its detection and, thus, it was widely disseminated throughout the herd where 77 % of the progeny of one individual carrier sow had inherited the translocation.

Generally, the detection of hypoprolific boars based on fertility data is only possible after a large number of litters are produced and by that time, an AI boar may have been used for more than four months and could serve hundreds of sows, leaving a large number of potential carrier descendants to be tested and identified. The DBE which is based on routine computations of the Canadian national swine breeding database has been increasingly used as an enhanced detection tool for hypoprolific boars [14, 35]. The four carriers, for which DBE values were available, all had values that were considerably lower than 0 (−4.82, −4.4, −4.39 and −2.67), which indicates the usefulness of this parameter; however, its calculation requires a minimal amount of mating data, which limits its practical application. Nevertheless, DBE is a valuable tool for screening relatives of an animal that was previously identified as a carrier of a chromosome abnormality, especially in cases where the parents of the carrier are no longer available for karyotype analysis to investigate the origin of the abnormality. In this study, cases 2, 5 and 11 exemplify the usefulness of the DBE parameter when the origin of the chromosomal rearrangements could not be confirmed. By reviewing the DBE values of related males, it was possible to speculate on the origin of the abnormality. However, much less is known about the specific rate of chromosome abnormalities according to the sex of the animals being bred. Here, we karyotyped both parents of five identified boar carriers and the maternal inheritance of the reciprocal chromosome translocation was determined in three of these cases, while two of the chromosome rearrangements occurred de novo. It should be noted that low fertility in the case of the sows’ performance (e.g. hypoprolificacy) may go undetected due to the number of parities needed for record analysis at the farms, unless fertility reduction is eventually quite large. Thus, it is expected that any rate of chromosome abnormalities observed in pig populations would include both origins, inherited and de novo chromosome rearrangements.

It is clear from the published case reports and large-scale population surveys that chromosome abnormalities are present in pig populations around the world and have a negative impact on reproductive efficiency and result in substantial economic losses [5, 27]. The extent of the financial loss is country- and farm-specific. In the context of this study, extrapolating the calculated frequency of 1.6 % of chromosome rearrangements to the commercial production scale, it is possible that over 46,400 of the approximately 2.9 million litters produced per year in Canada could be affected. At an average loss of four piglets per litter, the annual cost of piglets lost due to unidentified translocation carriers could be as high as $4.6 M.

## Conclusions

In this paper, we report the results of the largest systematic cytogenetic screening program for young breeding boars in Canada, to date. The results, in agreement with previous studies, underline the high incidence and variability of chromosome abnormalities in domestic pig populations. The prevalence of translocations in the Canadian pig populations (1.64 %) is higher than frequencies that have been reported in different countries. Reciprocal translocations seem to be the most frequent chromosomal abnormality occurring in domestic pig populations; they have a large negative effect on the fertility of carrier animals, which leads to smaller litter sizes and increased numbers of stillbirth and mummified piglets, as the most common in-farm recordable outcomes. Three out of five reciprocal chromosome translocations were maternally inherited, and two occurred as de novo chromosome rearrangements, which highlight the role of karyotype analysis as a laboratory test for elite genetic pigs of both sexes. Chromosome inversions and Robertsonian translocations have a smaller effect on the reduction in litter size of carriers. These findings emphasize the relevance of cytogenetic screening programs to systematically test all breeding boars as an essential tool for swine improvement in any country with an intensive pork industry.

## Additional file

10.1186/s12711-016-0246-5 Detailed description and representative karyotype images for the 11 cases analyzed in this study.
