# Supplementary material for: Prevalence and consequences of chromosomal abnormalities in Canadian commercial swine herds
Source: Genet Sel Evol. 2016 Sep 12;48(1):66. doi: 10.1186/s12711-016-0246-5 (PMC5018939; doi:10.1186/s12711-016-0246-5)

Additional files

Additional file 1.

The G-banded karyotypes were arranged according to the Standard karyotype of the domestic pig (Committee for the Standardized Karyotype of the Domestic Pig, 1988).

List of chromosome rearrangements:

Case 1: rcp(1;5)(q21;q23) identified in a Landrace boar

Case 2: rcp(1;15)(q211;q13) identified in a Yorkshire boar

Case 3: rcp(2;5)(p16;p11) identified in a Landrace boar

Case 4: rcp(3;4)(p15;q13) identified in a Landrace boar

Case 5: rcp(3;12)(p13;q15) identified in a Yorkshire boar

Case 6: rcp(6;7)(p15; q13) identified in a Duroc boar

Case 7: rcp(7;15)(q13;q13) identified in a Duroc boar

Case 8: rcp(8;13)(p21;q41) identified in a Landrace boar

Case 9: rcp(12;14)(q15;q23) identified in two Duroc boars

Case 10: Rob(13;17) identified in a Landrace boar

Case 11: inv(8)(q11;q25) identified in a Duroc boar

Additional Figure 1. Case 1: A Landrace boar, chromosome translocation carrier of rcp(1;5)(q21;q23).

GTG-banded karyotype revealed a reciprocal chromosome exchange between SSC1 and SSC5. SSC1 was shortened in its long arm (1q-) while SSC5 was lengthened in its long arm (5q+). Chromosome break points were located at the subterminal G-negative band of SSC1 and the terminal G-negative band of SSC5, the arrangement was defined as rcp(1;5)(q21; q13) translocation.

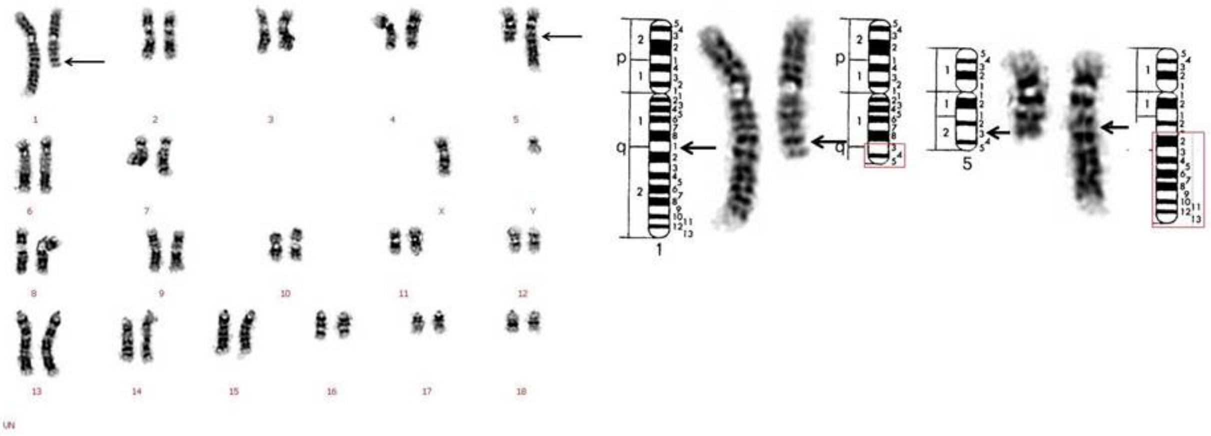

Additional Figure 2. Case 2: A Yorkshire boar, chromosome translocation carrier of rcp(1;15)(q211;q13).

GTG-banded karyotype revealed a reciprocal chromosome exchange between SSC1 and SSC15. SSC1 was lengthened in its long arm (1q+) while SSC15 was shortened in its long arm (5q+). Chromosome break points were located at the terminal G-negative band of SSC1 and the subterminal G-negative band of SSC15, the arrangement was defined as rcp(1;15)(q211;q13) translocation.

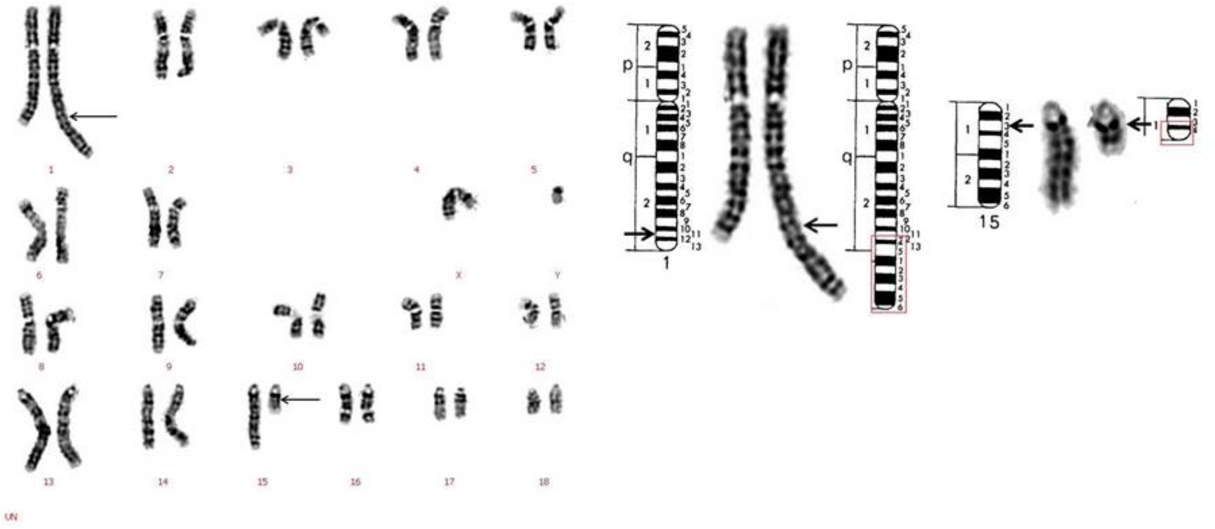

Additional Figure 3. Case 3: A Landrace boar, chromosome translocation carrier of rcp(2;5)(p16;p11). GTG-banded karyotype revealed a reciprocal chromosome exchange between SSC2 and SSC5. SSC2 was lengthened in its short arm (2p+) while SSC5 was shortened in its short arm (5q-). Chromosome break points were located at the terminal G-positive band of SSC2 and the interstitial G-negative band of SSC5, the arrangement was defined as rcp(2;5)(p16;p11) translocation.

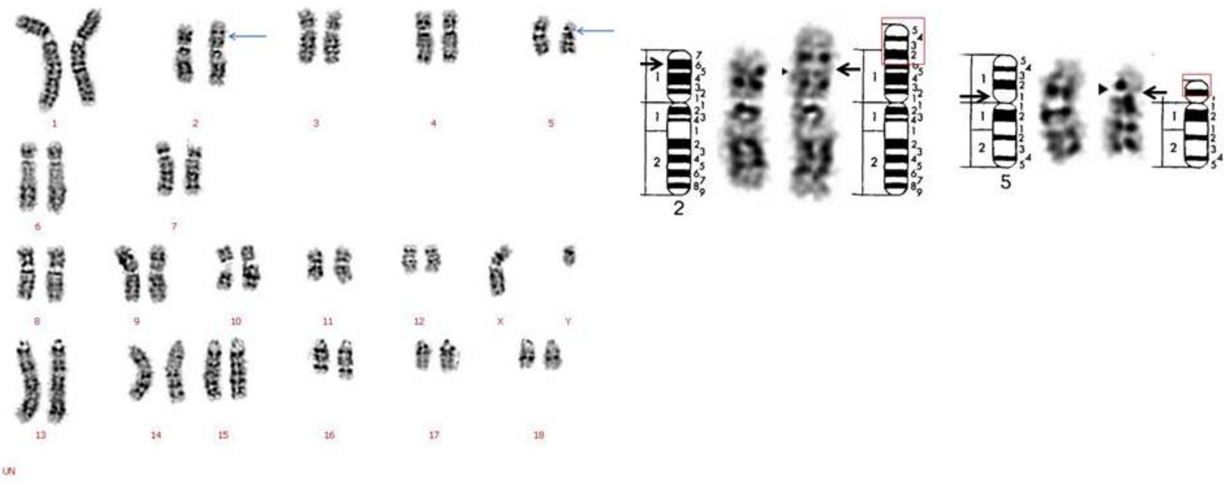

Additional Figure 4. Case 4: A Landrace boar, chromosome translocation carrier of rcp(3;4)(p15;q13).

GTG-banded karyotype revealed a reciprocal chromosome exchange between SSC3 and SSC4. SSC3 was lengthened in its short arm (3p+) while SSC4 was shortened in its long arm (4q-). Chromosome break points were located at the terminal G-negative band of SSC3 and the subterminal G-negative band of SSC4, the arrangement was defined as rcp(3;4)(p15;q13) translocation.

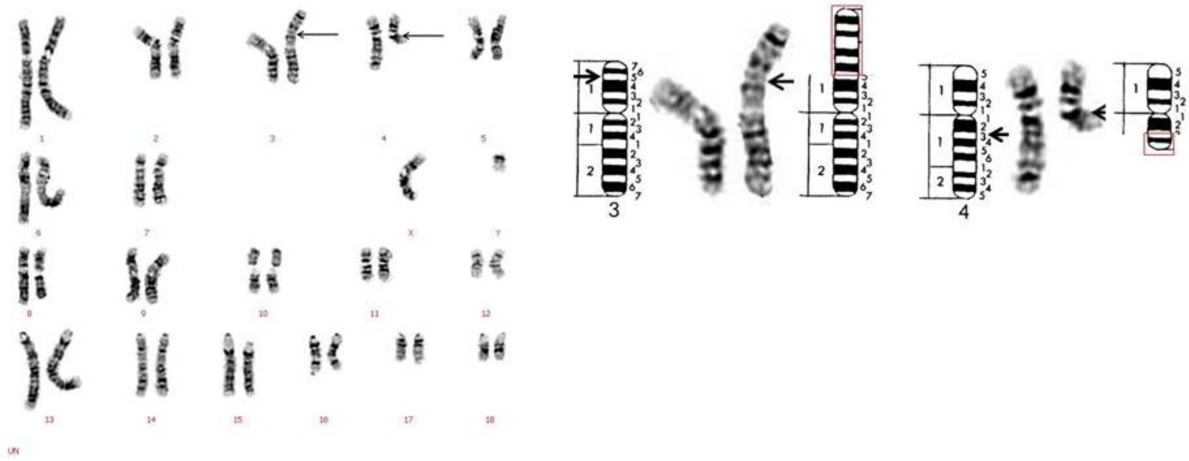

Additional Figure 5. Case 5: A Yorkshire boar, chromosome translocation carrier of rcp(3;12)(p13;q15).

GTG-banded karyotype revealed a reciprocal chromosome exchange between SSC3 and SSC12. SSC3 was shortened in its short arm (3p-) while SSC12 was lengthened in its long arm (12q+). Chromosome break points were located at the subterminal G-negative band of SSC3 and the terminal G-negative band of SSC12,the arrangement was defined as rcp(3;12)(p13;q15) translocation.

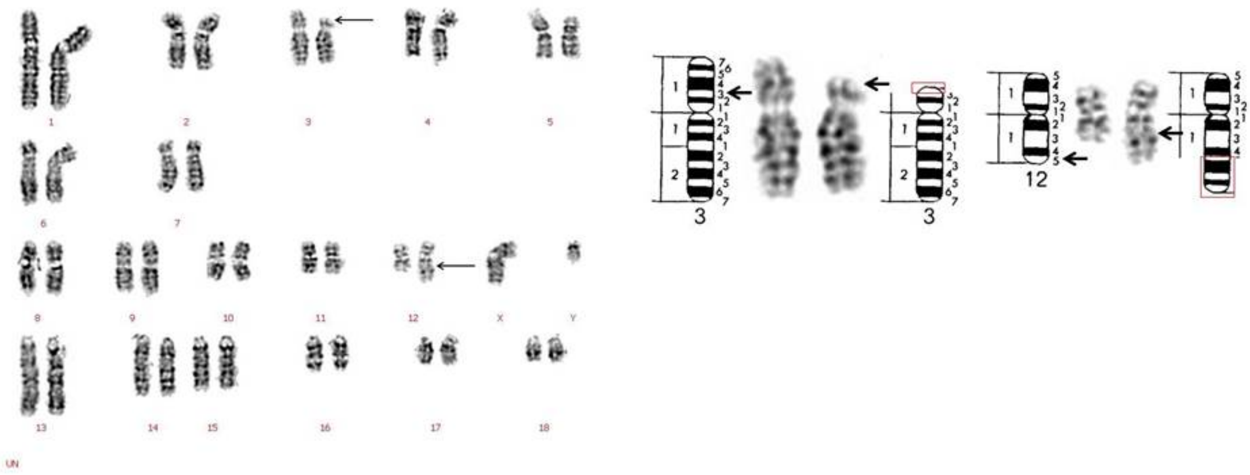

Additional Figure 6. Case 6: A Duroc boar, chromosome translocation carrier of rcp(6;7)(p15; q13).

GTG-banded karyotype revealed a reciprocal chromosome exchange between SSC6 and SSC7. SSC6 was lengthened in its short arm (6p+) while SSC7 was shortened in its long arm (7q-). Chromosome break points were located at the terminal G-negative band of SSC6 and the subterminal G-negative band of SSC7, the arrangement was defined as rcp(6;7)(p15; q13) translocation.

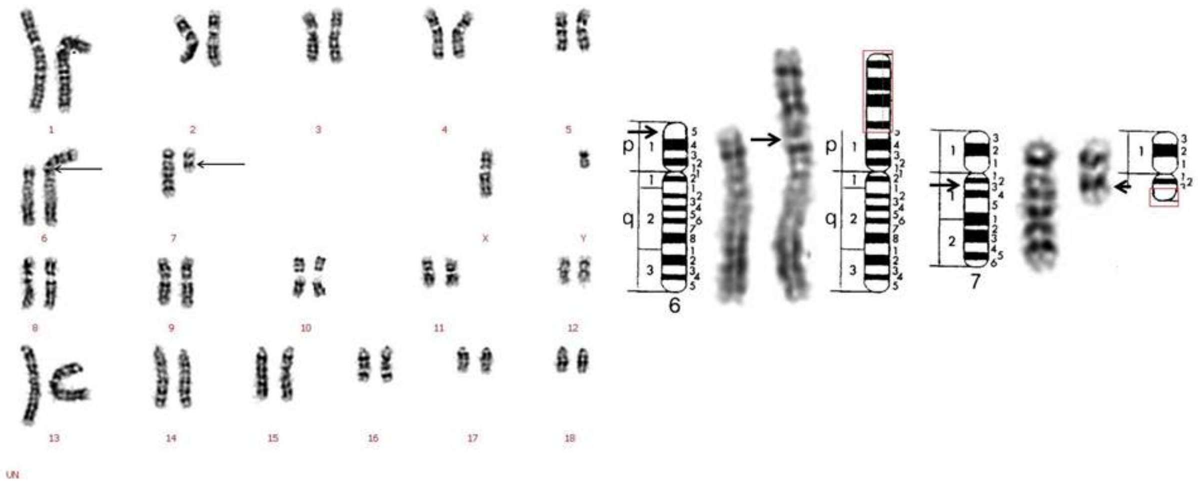

Additional Figure 7. Case 7: A Duroc boar, chromosome translocation carrier of rcp(7;15)(q13;q13).

GTG-banded karyotype revealed a reciprocal chromosome exchange between SSC7 and SSC15. SSC7 was lengthened in its long arm (7p+) while SSC15 was shortened in its long arm (15q-). Chromosome break points were located at the subterminal G-negative band of chromosome 7 and the subterminal G-negative band of SSC15, the arrangement was defined as rcp(7;15)(q13;q13) translocation.

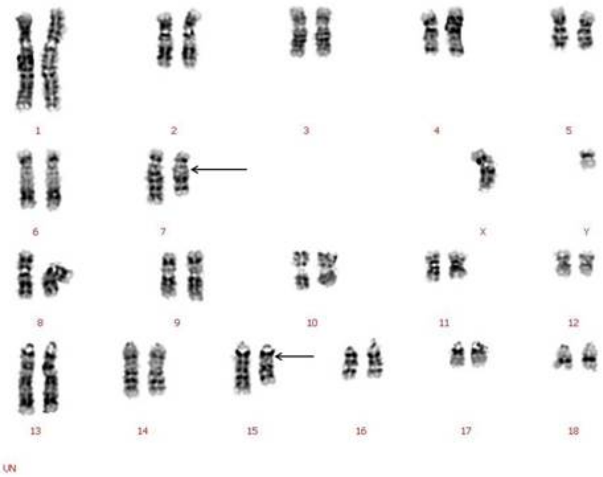

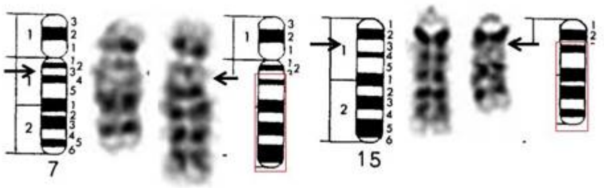

Additional Figure 8.Case 8: A Landrace boar, chromosome translocation carrier of rcp(8;13)(p21;q41).

GTG-banded karyotype revealed a reciprocal chromosome exchange between SSC8 and SSC13. SSC8 was lengthened in its short arm (8p+) while SSC13 was shortened in its long arm (13q-). Chromosome break points were located at the subterminal G-negative band of SSC8 and the subterminal G-negative band of SSC13, the arrangement was defined asrcp(8;13)(p21;q41) translocation.

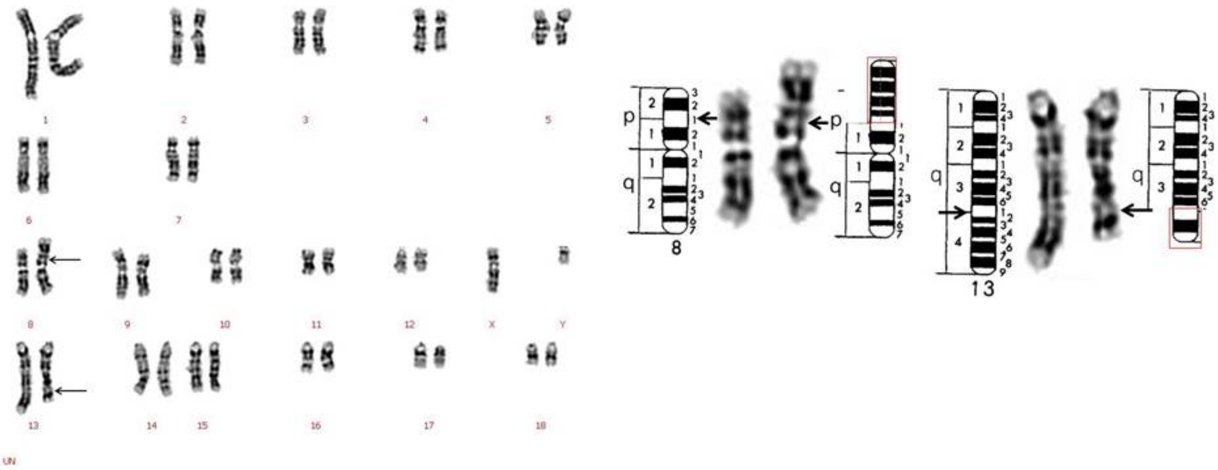

Additional Figure 9. Case 9: Two Duroc boars, chromosome translocation carriers of rcp(12;14)(q15;q23).

GTG-banded karyotype revealed a reciprocal chromosome exchange between SSC12 and SSC14. Most of SSC14 appeared translocated to the short arm of SSC12. Chromosome break points were located at the terminal G-negative band of SSC12 and the subterminal G-negative band of SSC14, the arrangement was defined as rcp(12;14)(q15;q23) translocation.

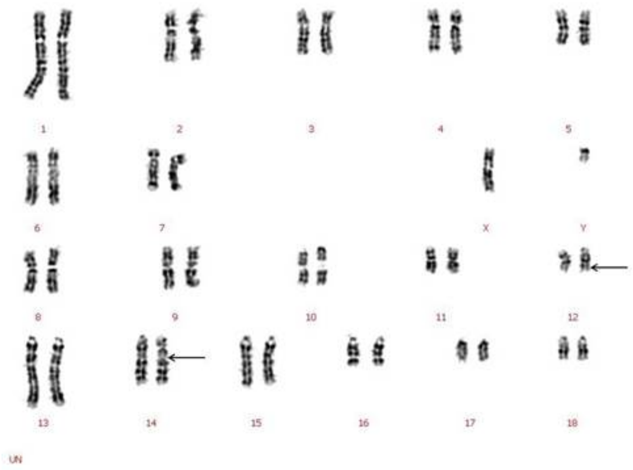

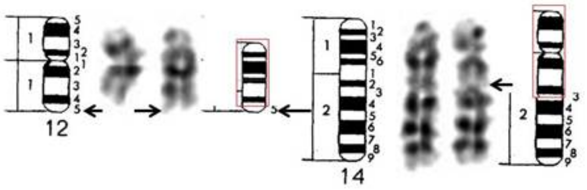

Additional Figure 10. 0: A Landrace boar, Robertsonian translocation carrier of rob(13;17).

The centromeric fusion of two acrocentric chromosomes, SSC13 and SSC17 resulted in the formation of an extra long submetacentric chromosome, reducing to 2n=37 the diploid number of the boar carrier Arrows indicate the derived chromosomes. (a) and (b): GTG banded karyotypes of boars carrying heterozygous and homozygous rob(13;17), respectively. (c): C band of a boar carrying Rob(13;17) in heterozygous form. (d) DAPI stain of a boar carrying Rob(13;17) translocation homozygous form.

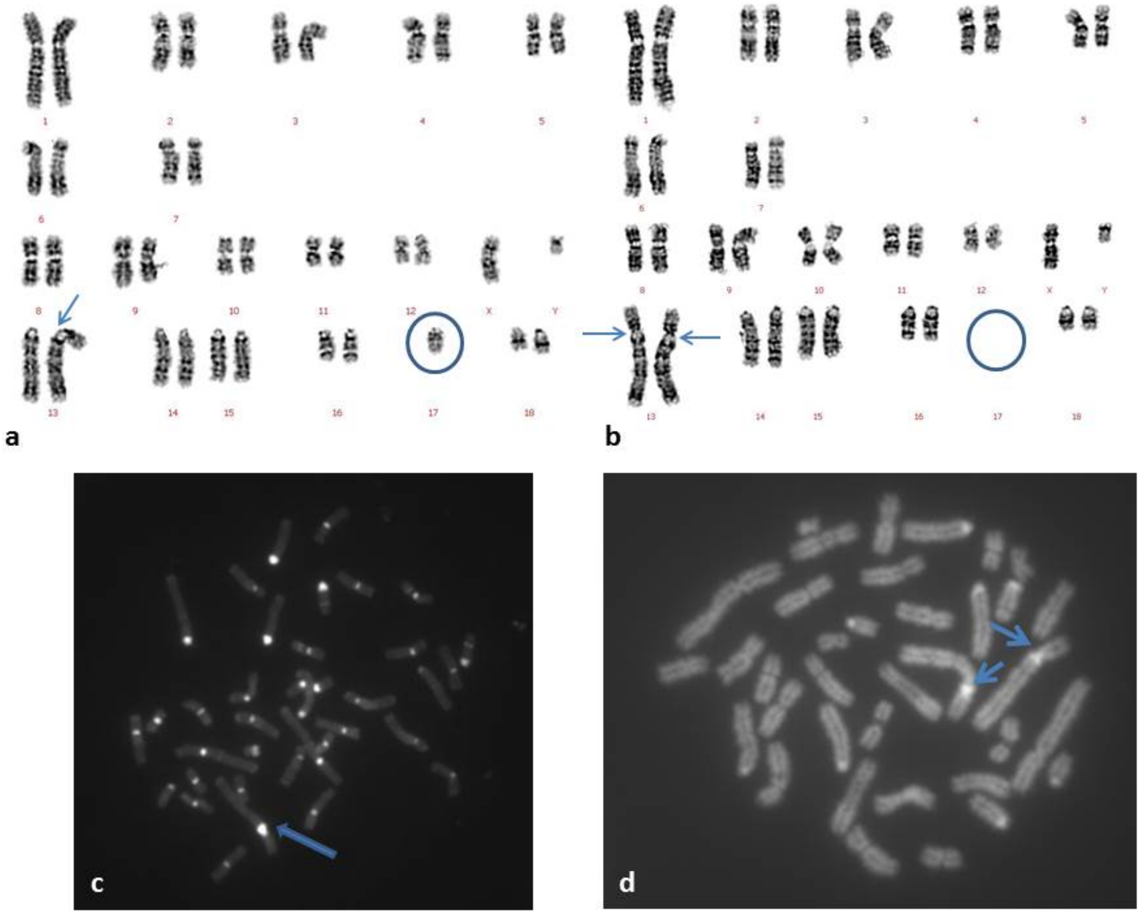

Additional Figure 11. Case 11: A Duroc boar, chromosome inversion carrier of inv(8)(q11;q25).

GTG-banded karyotype revealed a paracentric inversion of the long arm of SSC8. Chromosome break points were located at two distinctive G-negative bands (8q11 and 8q25) of inverted SSC8. The chromosomal rearrangement could be described as inv(8)(q11;q25) translocation.

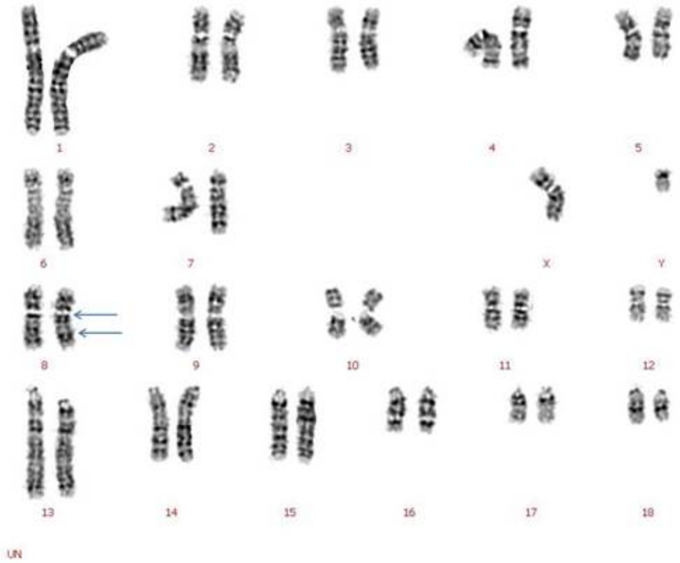

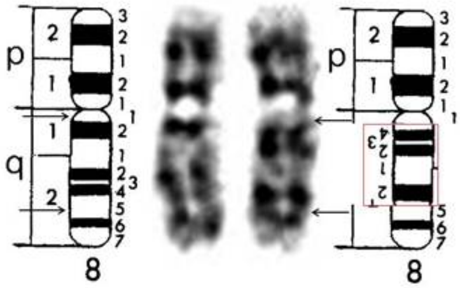

Supplement: Supplementary file 1 — 10.1186/s12711-016-0246-5 Detailed description and representative karyotype images for the 11 cases analyzed in this study. [file 12711_2016_246_MOESM1_ESM.pdf]
